# Marine Survival in the Mediterranean: A Pilot Study on the Cognitive and Cardiorespiratory Response to Sudden Cool Water Immersion

**DOI:** 10.3390/ijerph19031601

**Published:** 2022-01-30

**Authors:** Alex Buoite Stella, Shawnda A. Morrison

**Affiliations:** 1 Clinical Unit of Neurology, Department of Medicine, Surgery and Health Sciences, University Hospital of Trieste, University of Trieste, 34149 Trieste, Italy; 2 Centre for Climate Change and Active Children, Faculty of Sport, University of Ljubljana, 1000 Ljubljana, Slovenia; shawnda.morrison@fsp.uni-lj.si

**Keywords:** climate change, human migration, cold shock, heart rate, executive function, environmental epidemiology

## Abstract

Background and Aim: The Mediterranean is one of the major gateways of human migratory fluxes from Northern Africa, the Middle East, and Central Asia to Europe. Sea accidents have become an urgent humanitarian crisis due to the high number of migrants on the move, but data on the physiological effects to sudden cool water immersion are not as extensive as cold-water studies. We wanted to evaluate to what extent cool water immersion (~18 °C) may detrimentally affect cognitive ability and cardiorespiratory strain compared to the more prevalent cold-water (<10–15 °C) studies. Methods: In this case, 10 active, healthy men participated in this study which consisted of completing one familiarization trial, and then a control (CON) or experimental (EXP) trial in a randomized, repeated-measures, cross-over fashion, separated by at least 7-days. Cognitive function was assessed via the Symbol Digit Modalities Test (SDMT), a code substitution test, performed at baseline, then repeated in either a thermoneutral (~25 °C room air) dry environment, or when immersed to the neck in 18 °C water. Testing consisted of six “Step” time-blocks 45-s each, with a 5-s pause between each Step. Cardiorespiratory measures, continuously recorded, included heart rate (beats per minute), minute ventilation ($\dot{V}$_E_, L∙min^−1^), oxygen consumption ($\dot{V}$O_2_, L∙min^−1^), and respiratory frequency (fR, count∙min^−1^). Results: Initial responses to cool water (<2 min) found that participants performed ~11% worse on the code substitution test (*p* = 0.025), consumed 149% greater amounts of oxygen (CI: 5.1 to 9.1 L∙min^−1^, *p* < 0.0001) and experienced higher cardiovascular strain (HR CI: 13 to 38 beats per minute, *p* = 0.001) than during the control trial. Physiological strain was in-line to those observed in much colder water temperature. Conclusion: Sudden, cool water immersion also negatively affects cognitive function and cardiorespiratory strain, especially during the first two minutes of exposure. The magnitude increase in heart rate is strongly associated with poorer cognitive function, even in (relatively) warmer water consistent with temperatures found in the Mediterranean Sea environment.

## 1. Introduction

Accidental water immersion represents a common cause of death in many countries, with cold water representing a further risk due to the combination of drowning, cold shock response, and hypothermia [1,2,3]. For context, between 1978 and 1998 more than 5300 passengers were killed in ferry accidents around the world, making ferry travel 10 times more dangerous than air travel [4]. Cold water can be defined as water temperature less than 15 °C, and it represents an extremely hazardous scenario, physiologically speaking, since tachycardia, hyperventilation, tachypnea, increased blood pressure, and possible arrhythmias are likely with sudden, cold-water immersion [5,6]. In 1981, Golden and Harvey identified four stages of water immersion, each associated with their own specific risks. These include: (i) initial responses (first 3 min), (ii) short term responses (over 3 min), (iii) long term responses (over 30 min) and (iv) post immersion/circum-rescue responses [6]. In particular, the initial cold shock phase seems to be responsible for the majority of immersion deaths, in part due to a strong gasp response and uncontrollable hyperventilation [7,8]. Most research studies investigating the initial responses to cold shock have (rightly) been focused on cold or very cold-water temperature scenarios, due its severe and immediate impact on health and survival [6]. However, physiological responses to sudden water immersion may also be present in warmer water, perhaps even up to 25 °C, and especially for those who are not acclimated to sudden water immersion.

The sea surface temperature (SST) in the Mediterranean Sea, measured at 4 m depth, is usually warmer than in the oceans and water surrounding Northern Europe. In 2006, the Western Mediterranean recorded an annual mean SST of ~19 °C, whilst for the Eastern Mediterranean, it was even warmer (~21 °C) [9], with noted rising temperature trends present for the last few decades. The sea temperature tends to oscillate between winter and summer by ±4 °C [10]. Throughout the year, the Mediterranean represents one of the major gateways for human migratory fluxes from Northern Africa, the Middle East, and Central Asia to Europe. Indeed, sea accidents have become an urgent humanitarian crisis, due to the especially high number of migrants on the move [11]. This includes at least 1146 people who have died attempting to reach Europe by sea in the first six months of 2021, constituting a substantial increase compared to fatalities reported in the same period in 2020 (513) and 2019 (674) [12]. These human migration patterns are expected to persist, especially with the ever-increasing environmental pressures extolled by climate change, itself bringing high heat events, drought, wildfire, food and water shortages, and civil unrest [13].

One of the greatest survival challenges occurring in the initial phase of sudden water immersion is making the best outcome decision in a high-stress, dynamic situation, e.g., deciding whether to “stay or swim” [14], and therefore, maintaining adequate cognitive performance plays a central role in determining one’s own survival chances. During the initial responses to cold-water immersion, physiological functions are altered immediately, and these responses are often detrimental for the possibilities of rescue. Results from previous work suggest that immediately after cold-water immersion, mental performance and visual function are reduced, and working memory is negatively affected during sea survival scenarios, as previously reported [15,16,17,18,19]. Thus, the aim of this study was to evaluate to what extent sudden exposure to cool water immersion (~18 °C) in unacclimated humans may elicit the same (or similar) cognitive and cardiorespiratory effects as have been observed in cold-water studies, better reflecting the nature of the Mediterranean Sea environment. It was hypothesized that physiological variables would be negatively affected in the warmer water, and related to overall changes in cognitive function.

## 2. Materials and Methods

### 2.1. Participants and Study Design

Ten healthy, male participants were recruited to participate in the study (age: 18–35 years) who reported no history of repeated cold exposure or cold-induced illness (e.g., Raynaud’s disease). Exclusion criteria were body mass index (BMI) > 30 kg/m^2^, cognitive impairment, previous history of cardiovascular, respiratory, neurological, psychiatric, and skin diseases. All participants were instructed about the aim and protocol of the study, and signed a written, informed consent before inclusion to the study. This study was approved by the institutional review board and ethical committee of the Trieste University Hospital. All participants performed a familiarization/training session plus two experimental conditions, each separated by at least seven days. Participants underwent both experimental conditions following a randomized, repeated-measures, cross-over design. Randomization was obtained by tossing a coin for each participant, with one face of the coin corresponding to a sequence of experimental sessions. When half of the participants (5) were allocated to a sequence, the remaining participants were automatically allocated to the other one. During the familiarization/training session a physician assessed participants’ health status with an anamnestic check-up and electrocardiography (ECG)/blood pressure (BP) at rest and after a short, three-minute stepping effort.

### 2.2. Experimental Protocol

One week before the first experimental session, participants were invited to the research facility and received a training copy of the Symbol Digit Modalities Test (SDMT), a code substitution test. After medical examination, participants’ demographics and anthropometrics were recorded. Room air temperature was measured with a digital thermometer (Rocktrail IAN 58787, Bedfordshire, UK). The control condition (CON) was performed in a dry thermoneutral environment (25.6 ± 0.9 °C), whilst the experimental cool water immersion condition (EXP) was performed via a head-out full-body immersion in a portable bath (Qryo srl, Fagagna, Italy), with water mildly stirred, and constantly maintained at ~18 °C (18.5 ± 0.4 °C) by a pump and refrigerating system connected to the bath. Water temperature was constantly monitored by a digital thermometer that was part of the refrigerating system connected to the portable bath. Trials were performed at rest, at least two hours after the participants woke up to avoid any lingering effects of their sleep inertia. No alcohol, coffee, tobacco, or carbohydrates were consumed at least 1 h prior to measurements. Participants were asked to reach the research facility 60 min before the start of the measurements to acclimate to the environment, wearing only a t-shirt and short swim trunks for both conditions.

**Figure 1:**
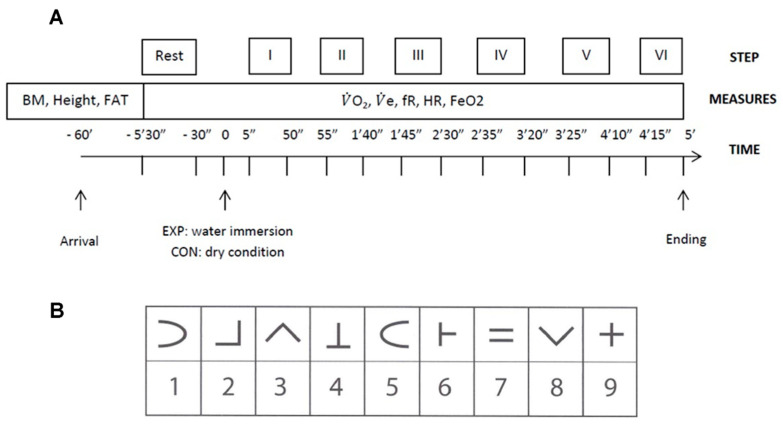
Timeline of the experimental session, including (**A**) the Step sequences and (**B**) an example of the SDMT matrix. Note: The full SDMT test depicts a printed matrix on one sheet of paper that includes a random sequence of 45 symbols and blank squares. Each square is completed by the participant who either writes their answer into the square (on land), or verbally indicates their answer to the researcher, who immediately records the result (water trials).

Baseline cardiorespiratory parameters were collected for 5 min whilst participants sat on a chair (BL). During the last 30 s of BL, participants performed the cognitive test warm up. After completing this task, during the CON trial, participants were asked to perform the SDMT while remaining on the chair, whereas during the EXP trial, participants entered the bath and were seated on a stool, with water reaching the level of their collarbone. Cognitive and cardiorespiratory parameters were continuously collected for five minutes. The five minutes of measurements were divided into six steps of 45-s (with a 5-s pause between each step); Steps corresponded to using a different SDMT sheet, and these same epochs were used to average physiological responses within that timeframe (Step I, II, III, IV, V, VI, Figure 1A).

### 2.3. Cognitive Function

The Symbol Digit Modalities Test (SDMT), a code substitution test, was used to assess cognitive function. The test is a reliable and repeatable method used in neuropsychology to assess information processing speed, selective attention and working memory [20]. This test is widely used in clinical settings to assess cognitive functions in patients with dementia or other neurological diseases, but it has also been used in experimental studies looking at the environmental effects on mental performance [16,17,21,22,23,24]. It can be proposed in both the oral and written version, the latter influenced by manual dexterity and coordination of the participant. In detail, the SDMT consists of a sheet of paper with a matrix of nine symbols and nine corresponding numbers at the top (Figure 1B). On the same sheet, below the matrix, there is a sequence of symbols with a blank square where it is possible to write the corresponding number (as fast as possible). The standard protocol consists of matching a maximum of 110 symbols in 90 s. In the present study, we used an adapted, halved-time version consisting of 45-s for every sheet instead of the typical 90-s to increase the temporal resolution. In this case, 10 sheets were printed with a random matrix on the top and a random sequence of 55 symbols with corresponding blank squares on the bottom. Participants could look at the matrix during the entire measurement session. After an initial warm up phase was included where participants were asked to match 10 symbols without any time pressure, the official testing began. Participants had 45-s to match as many symbols as possible, with a maximum of 55 symbols for each sheet available to complete. After each 45-s time-block, a new sheet was produced, showing a completely different matrix, a novel 55 symbol sequence was provided, and so on. Measurements started 5-s after the participant was immersed (or in the control condition, 5-s after indicating the test will begin). There was a 5-s pause after each step until the end of data collection, which occurred at the 5-min mark. One experimenter would reveal the SDMT sheet to the participant whilst another experimenter recorded results on separate copy of the identical test sheet. In both trials, results were conveyed by the participant orally to the researchers. Results were recorded for each Step to obtain a time-sequence evaluation of cognitive performance. Test outcomes were the SDMT score (i.e., the number of correct combinations symbol-number) and the number of errors for each sheet/step individually.

### 2.4. Anthropometry and Cardiorespiratory Measures

Body mass (kg), height (m) and body fat (bf%) were determined during the familiarization session. Percent body fat was estimated using a bioimpedance device according to the manufacturer’s instructions (Handy3000, DS Medica Srl, Milan, Italy). Body surface area (BSA, m^2^) was calculated using the DuBois and DuBois equation [25]. Cardiorespiratory parameters were measured and recorded using a metabolic cart (Fitmate Plus, Cosmed Srl, Milan, Italy) calibrated according to manufacturer’s instructions. The system measures oxygen uptake (at rest or during exercise) with the same accuracy of conventional systems with both O_2_ and CO_2_ sensors, but provides an easier test process (no calibration with gas cylinders, no warm-up), although it does not provide CO_2_ data [26]. Heart rate (HR, bpm) was continuously recorded using a Polar heart rate monitor (Polar, Sweden) interfaced to the metabolic cart via wireless receiver. Maximum heart rate (%HRmax) was estimated using the Tanaka et al. equation [27]: HRmax (bpm) = 208 − 0.7 × age. Heart rate range (HRR) was then defined as the difference between HRmax and HR at rest, and the percentage of HRR was then computed for each step. Minute ventilation ($\dot{V}$_E_, L∙min^−1^), oxygen consumption ($\dot{V}$O_2_, L∙min^−1^), and respiratory frequency (fR, count∙min^−1^) and exhaled fraction of oxygen (FeO_2_, %) were recorded continuously throughout the trials.

### 2.5. Statistical Analysis

All data were analysed using the statistical package IBM SPSS Statistics for Windows, Version 27.0. (Armonk, NY, USA, IBM Corp.). As mentioned previously, all physiological continuous data were averaged within each 45-s measurement step. Change scores between trials were calculated within each step for any given dependent measure (e.g., heart rate or oxygen consumption). Differences in laboratory ambient temperature were analysed using a pairwise *t*-test, at the *p* < 0.05 level of significance. For cognitive and physiological dependent measures, a two-way repeated measures ANOVA was run to determine whether any differences existed between time-points (step levels) and/or trial (two levels: control, experimental cool water immersion). When a significant main or interaction effect was observed, *t*-tests were conducted in post-hoc fashion as needed. Significance was determined at the *p* < 0.05 level of significance. Means ± standard deviations are given in the text and tables, with 95% confidence intervals (CI) provided where appropriate for interest.

## 3. Results

### 3.1. Participants

Ten healthy young males (26.7 ± 4.4 years) participated in the study (Table 1). Ambient conditions of the testing location were not different between trials (CON: 25.6 ± 0.8 °C, EXP: 26.4 ± 1.1, *p* = 0.07).

### 3.2. Cognitive Function

**Figure 2:**
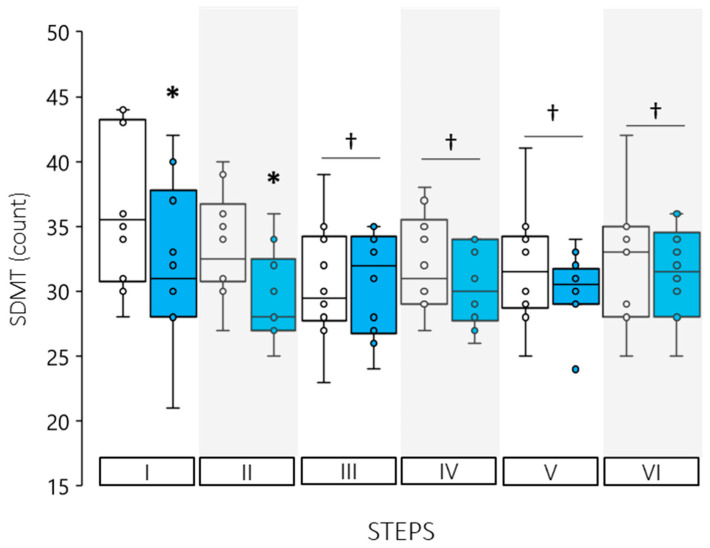
SDMT results for control (open boxes) and experimental trials (shaded boxes). (*) Indicates a significant difference between trials for that Step, (†) Indicates a significant time main-effect difference from Step I (*p* < 0.05).

There was a significant time by trial interaction effect (F = 2.442, *p* = 0.048) for changes observed in SDMT performance such that cognitive function was lower immediately following sudden cool water immersion (Step I: CON: 36.1 ± 5.8, EXP: 32.1 ± 6.2, CI: 0.6 to 7.4 count, *p* = 0.025), and persisted for the first two minutes of immersion (Step II: CON: 33.4 ± 4.1 EXP: 29.4 ± 3.5, CI: 0.9 to 7.1 count, *p* = 0.016, Figure 2). Thereafter, SDMT performance plateaued for the remainder of the trial, such that from Step III to Step VI results were lower than the first two Steps, and not different between trials. The error count for CON and EXP moving through Step I to Step VI were: 1, 0, 0, 2, 0, 2, and 3, 3, 0, 0, 2, 0, respectively. Thus, within the first 2 Steps there were 5 more errors made compared to the CON trial.

### 3.3. Cardiorespiratory Strain

**Figure 3:**
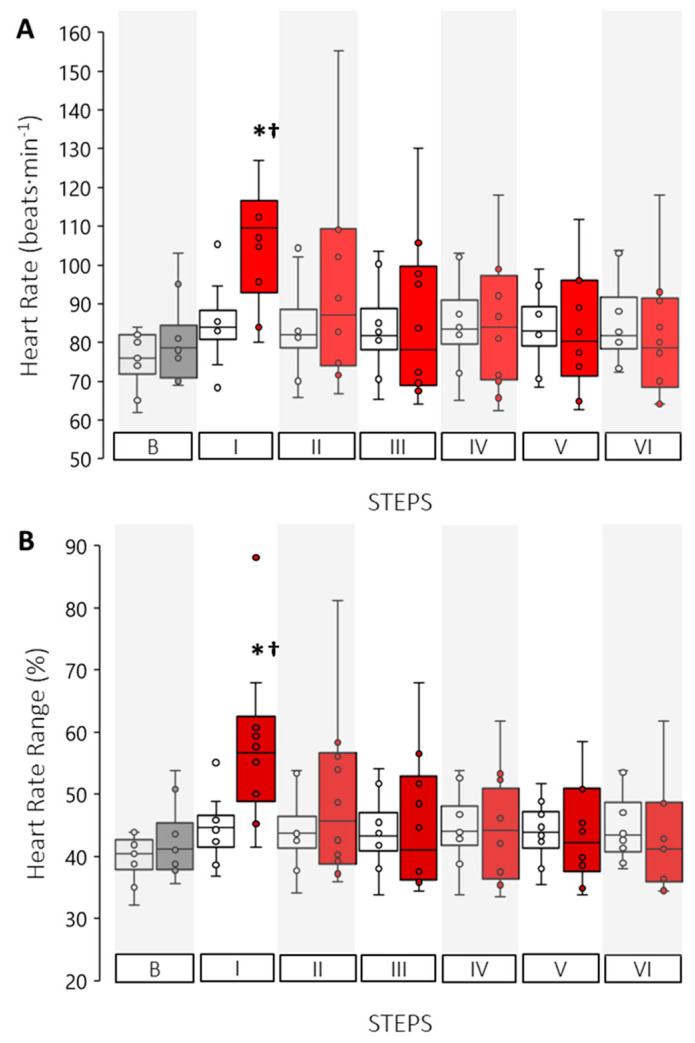
Cardiovascular strain results for control (open boxes) and experimental trials (shaded boxes) for (**A**) heart rate and (**B**) percent heart rate maximum. (*) Indicates a significant difference between trials for that Step, (†) Indicates a significant time main-effect difference from all other Steps (*p* < 0.05).

Changes in HR to water immersion demonstrated a significant interaction effect (*p* < 0.0001) such that upon initial exposure to cool water, participants’ cardiovascular strain increased ~28% from baseline (Figure 3A) and an absolute increase of 26 bpm (CI: 13 to 38 bpm, *p* = 0.001) over the control trial at the same Step. This increase corresponded to a jump in relative heart rate range from baseline values of ~40% to upwards of ~60% HRR (CI: 7 to 20%, *p* = 0.001). Although heart rate data between trials was not different as the cool water exposure continued, there was greater variability in the data, suggesting some participants’ cardiovascular strain remained elevated throughout the 5-min test protocol.

**Figure 4:**
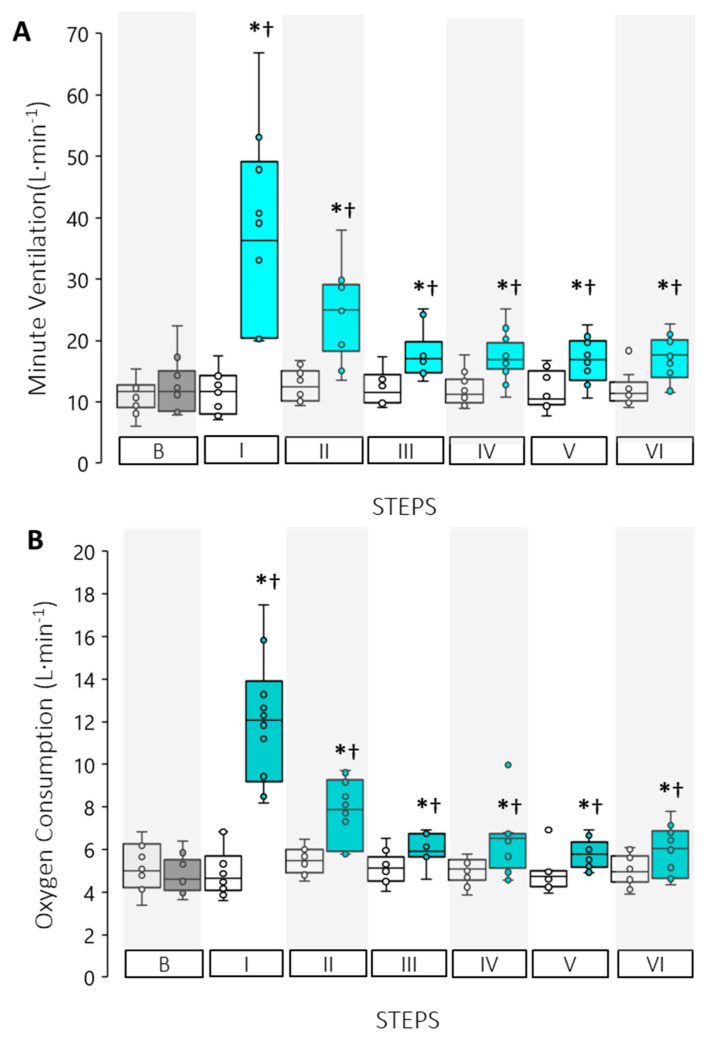
Respiratory results for control (open boxes) and experimental trials (shaded boxes) for (**A**) minute ventilation $\dot{V}$_E_, and (**B**) oxygen consumption $\dot{V}$ O_2_. (*) Indicates a significant difference between trials for that Step, (†) Indicates a significant time main-effect difference from Baseline (*p* < 0.05).

Minute ventilation ($\dot{V}$_E_, L∙min^−1^), demonstrated a significant interaction effect (*p* < 0.0001) during which the EXP trial observed significant increases from baseline to the Step I (12.4 ± 4.6 to 37.5 ± 15.5 L∙min^−1^, *p* < 0.0001). $\dot{V}$_E_ remained elevated compared to CON trial throughout the water immersion protocol (*p*-value range from 0.000 to 0.005, Figure 4A). Oxygen consumption ($\dot{V}$O_2_, L∙min^−1^) was significantly elevated from 4.8 ± 0.9 L∙min^−1^ at baseline to 12.1 ± 3.0 L∙min^−1^ in Step I (*p* < 0.0001), remaining elevated to the end of the trial (*p*-values ranging from 0.000 to 0.038, Figure 4B).

Exhaled fraction of oxygen (FeO_2_, %) was increased throughout the EXP trial compared to CON (*p* = 0.016) such that 95% CI indicate it remained ~0.1 to 1.3% higher than the CON for any given Step. Thus, FeO_2_ data collapsed between Steps, was 28.8 ± 9.9% for CON and 32.3 ± 10.3% for EXP, respectively. Respiratory frequency (fR, count∙min^−1^) was significantly elevated for both trials compared to baseline (*p* < 0.0001), moving from baseline values of 15.1 (CI: 12.9 to 17.3 count∙min^−1^) to a grand mean of 28.3 (CI: 22.7 to 34.0 count∙min^−1^) during experimental testing (range: 27.3 to 38.0 count∙min^−1^). Only in Step I were there differences between trials (CON: 29.5 ± 10.3 and EXP: 38.0 ± 8.9 count∙min^−1^, CI: 2.6 to 14.4, *p* = 0.010).

### 3.4. Correlations

**Figure 5:**
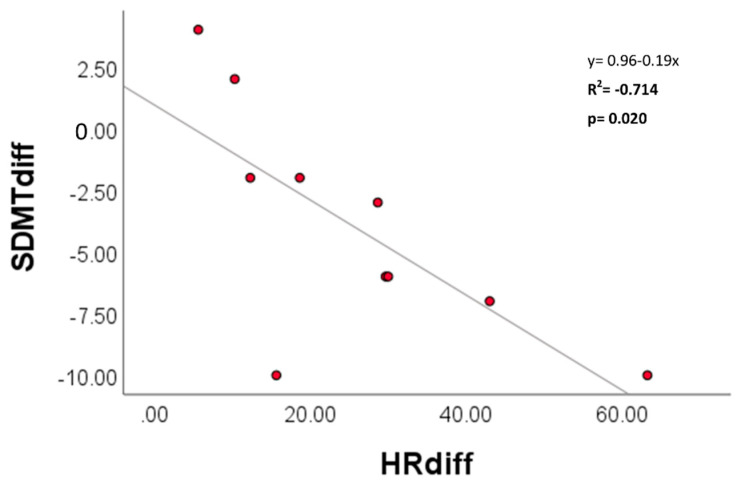
Significant correlations in change score between trials at Step I for SDMT and heart rate (HR).

Both the change in HR and change in %HRR were significantly correlated to poorer cognitive function, observed in SDMT Step I scores between trials (Figure 5). The strength of the linear relationship was strong, accounting for ~71–72% of the variance observed in variables between trials. No respiratory variables met the a priori level of significance.

### 3.5. Comparison to Cold Water Immersion

**Figure 6:**
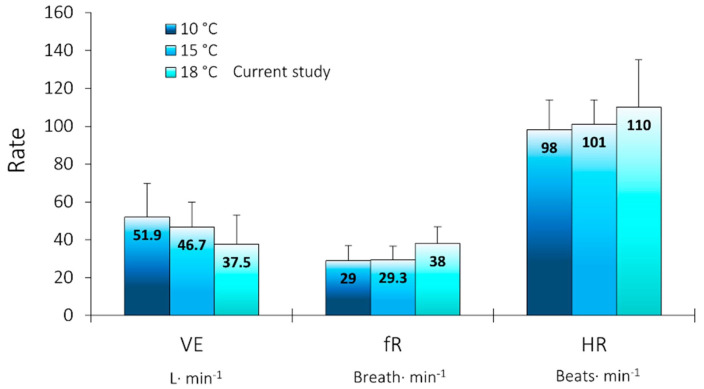
Comparison of key study results to cold-water literature at 10 °C [29]; and 15 °C [28] for minute ventilation (E), breathing frequency (fR), and heart rate (HR). Data presented are means plus standard deviation error bars, retrieved from the published literature for interest.

Comparing the initial cardiorespiratory cool water-shock responses (i.e., the Step I values) to those found elsewhere in the literature [28,29], our initial changes in minute ventilation were slightly lower than those observed in colder, 10 °C or 15 °C water, and both fR and HR were slightly higher, but all measures were within the standard deviation reported in the published studies (Figure 6).

## 4. Discussion

When accidents occur in a marine environment, cause of death can be complicated to ascertain, especially since multiple factors are often involved, and can include: the sudden, intense, immediate changes in cardiorespiratory response, the risk of drowning per se, and hypothermia. As such, the person at risk of drowning needs to be aware that making appropriate decisions quickly will increase their chances of survival [30]. For example, the increased physiological responses consequent to cold-shock reduce swimming efficiency, which initiates early muscular fatigue [14]. During cruises, it is beneficial to impart some general recommendations to the client, delivered as part of a mandatory process onboard the vessel. During an accident, however, such information might be confusing or inappropriately applied in practice. One of the main choices someone has to consider after falling into the sea is whether to “swim or stay”. The decision of remaining stationary and wait for help to arrive (thus avoiding the exertion of swimming) depends on several factors, including the estimated distance needed for self-rescue, water and air temperature, and swimming ability. This kind of decision should be postponed until after recovering from the initial cold shock which happens within the first three minutes of sudden water immersion [31]. Although the mechanism(s) underlying improper decision-making during maritime disasters are likely to include anxiety and panic, it is hypothesized that altered physiological responses (namely, cardiovascular and respiratory elevations) also have a role to play in the reduced cognitive capacity observed. In particular, this study showed that executive functions are compromised during the first 2–3 min of head-out cool water immersion in 18 °C water. Previous findings have shown that even warm water immersion might induce a small but significant increase in plasma catecholamines [32,33], which have been found to be potentially associated to poor cognitive function [34]. Nonetheless, the fast effect on cognitive functions found in this study suggest a potential role of other factors. A possible explanation as to why we observed this finding in a study conducted in a safe environment with limited (presumed) psychological stress could be because of the effect of hyperventilation on brain oxygen supply [35]. Indeed, previous findings suggest that hyperventilation per se can result in reduced oxygenated hemoglobin in the prefrontal cortex, which is the brain area dedicated to executive function [36].

The mechanisms underlying cardiorespiratory responses to cold-water immersion have been widely described, suggesting the contribution of skin cooling, anxiety and stress components, deep body cooling, and overriding conscious and autonomic cardiorespiratory controls [5]. Due to the nature of the temperature-dependent physical responses to water immersion [37,38], we argue that externally-valid water temperatures should be used to simulate real-life conditions. Indeed, it might be speculated that a different water temperature might affect the cooling rate of the skin, reflecting on different peripheral cold receptors activations, and therefore, different cardiorespiratory responses [5]. Cool water temperatures should be used when the aim is to understand the responses to sudden water immersion in warmer seas, such as the Mediterranean. This study found that immersion in cool water of 18 °C also resulted in a significant and rapid increases in all cardiorespiratory parameters, including minute ventilation, respiratory frequency, oxygen consumption and heart rate. In particular, ventilation, oxygen consumption and heart rate were found increased for all the duration of the 5 min immersion, which is in line with previous literature suggesting that the initial responses after cold shock remains elevated for about 3 min after initial exposure [5,28,29]. Among the respiratory responses, the respiratory frequency showed the fastest adaptation rate, with values being comparable to the dry thermoneutral control condition already after the first minute. Taken together, results from this study suggest a faster cardiorespiratory adaptation rate (as percentage of the difference between the first 30 s of immersion and between 30 s and 180 s) in 18 °C cool water immersion compared to 10 °C cold water immersion [29,39].

Heart rate reached a peak of about 60% of the estimated maximum heart rate range for each subject; however, the present study was performed with participants calm, at rest; they did not need to swim or float, so increases in this measure were reflecting the independent effect of water immersion and temperature stress. In the case of a sudden water immersion in a real-life context, (such as during a shipwreck, accident or capsize), anxiety, panic and the physical demands of escaping the vessel will further increase the cardiorespiratory demand, resulting in cardiac events, in particular in those with pre-existing medical conditions [6]. Increased ventilatory responses, which depend on the cold shock response and the increased request of the cardiac and respiratory muscles (as expressed by the increased oxygen consumption), represent a major risk for people during a sea survival situation, which are known to decrease maximal breath-hold time and increase the chances of ingesting small, but lethal, volumes of water [5].

The increased cardiorespiratory responses observed in warmer water consistent with the inner seas of the Mediterranean, highlight the importance of considering such factors when addressing health risk for a wide range of people, as seafarers, fishermen, and tourists. Additional training and education should be provided to these target people, emphasizing the importance of considering the timeline for self-rescue, and recognizing the different phases of initial cold shock responses to both cognitive and physiological parameters [31]. Vulnerable persons embarking on the dangerous marine crossings via the Mediterranean to reach Europe often consist of poor and unsecure ship conditions. Many humans fall into the water when these vessels leak or capsize, resulting in a humanitarian crisis due to the need for rescue and fatalities [11]. A high proportion of these migrants crossing the sea might be characterized by poor health and fitness status, might be malnourished and/or poorly clothed. A high proportion of migrants, in particular children, might also be unable to swim [40,41]. Together with poor clothing and the absence of life jackets, these factors certainly contribute to a higher risk of drowning even before hypothermia occurs. Taken together, the findings of the current study re-enforce the fact even under the most conservative scientific approach (safe environment, no physical exertion, relatively warmer water) initial cold-shock responses are physiologically relevant, and that sudden cool water immersion affects cardiac frequency, which itself can lead to an increased risk of drowning.

## 5. Study Considerations and Future Work

Unfortunately, due to the preliminary nature of the study it was not possible to perform direct measures of core or skin temperature in the hospital environment, thus we cannot comment on this important thermal input. We did not perform a thermoneutral water trial, and consequently there may be underlying hydrostatic issues that are not comparable between trials, including any fluid shifts brought on by the external pressure of the water, differences in blood flow to the organs (including the brain), or perceptual effects of water immersion on the skin surface. Despite this study found increased cardiorespiratory responses during immersion in a cool water condition, to what extent such responses might represent a realistic risk for a person being immersed remain to be elucidated. Future studies should aim to define the optimal responses for a decreased risk of water ingestions due to hyperventilation, for example, as this might help to identify the water temperature at which such intensity of the responses can be observed. Data were obtained only from male participants, and it is acknowledged that female participants and younger or older participants may have different responses to these stimuli, and the authors explicitly encourage future work in this area. The target population of this study are likely to be in poor nutritional habitus, therefore a food/sleep deprivation arm, taking place in an actual outdoor environment (e.g., waves, holding onto a surface) are being considered for future work in this area. Although the sample size of this study is relatively low, it is in-line with research from the field, and since we anticipated that most cognitive and physiological responses would be occurring in a uni-directional fashion, there was enough statistical power to determine whether the protocol would be able to adequately test our primary hypotheses. Future work should consider more insightful measures of cardio- and cerebrovascular function changes to the cool water stimulus, including whether any changes in cognitive function are associated with altered cerebral blood flow velocity or oxygenation levels, and to whether (or to what extent) cool water immersions may elicit autonomic conflict, especially for vulnerable populations (e.g., older adults) or unacclimated individuals.

## 6. Conclusions

Cold shock after cold water immersion provokes several dramatic physiological responses, especially in non-acclimatized people. These initial cardiorespiratory responses are also observed in ~18 °C cool water immersion, a temperature more comparable to the Mediterranean Sea. The magnitude and duration of physiological responses in the present study are not as extreme as those observed during cold water immersion (e.g., 10 °C or 15 °C). However, and despite the conservative research design used here, significant increases in both cardiovascular and respiratory strain are noted. Data confirm that the magnitude increase in heart rate is strongly correlated to poorer cognitive function, especially during the initial phase of cool water immersion.

## Figures and Tables

**Table 1:** Descriptive statistics of the study participants.

	N	Minimum	Maximum	Mean	Standard Deviation
Age (y)	10	20.0	32.0	26.4	4.2
Body Mass (kg)	10	63.0	85.0	75.1	7.6
BM kg∙m^−2^	10	20.3	25.6	23.5	1.8
BF (%)	10	11.0	22.0	16.2	3.4
BSA (m^2^)	10	1.76	2.13	1.93	0.12
T_water_ (°C)	10	18.0	19.0	18.5	0.4

## Data Availability

Data are available upon reasonable request to the corresponding author.

## References

[1] Massey H., Leach J., Davis M., Vertongen V. (2017). Lost at sea: The medicine, physiology and psychology of prolonged immersion. Diving Hyperb. Med. J..

[2] Schmidt A.C., Sempsrott J.R., Hawkins S.C., Arastu A.S., Cushing T.A., Auerbach P.S. (2019). Wilderness Medical Society Clinical Practice Guidelines for the Treatment and Prevention of Drowning: 2019 Update. Wilderness Environ. Med..

[3] Xu X., Giesbrecht G.G. (2018). A new look at survival times during cold water immersion. J. Therm. Biol..

[4] Faith N. (1998). Mayday: Disasters at Sea.

[5] Datta A., Tipton M. (2006). Respiratory responses to cold water immersion: Neural pathways, interactions, and clinical consequences awake and asleep. J. Appl. Physiol..

[6] Tipton M.J., Collier N., Massey H., Corbett J., Harper M. (2017). Cold water immersion: Kill or cure?. Exp. Physiol..

[7] Barwood M.J., Corbett J., Tipton M., Wagstaff C., Massey H. (2017). Habituation of the cold shock response is inhibited by repeated anxiety: Implications for safety behaviour on accidental cold water immersions. Physiol. Behav..

[8] Tipton M. (2003). Cold water immersion: Sudden death and prolonged survival. Lancet.

[9] Nykjaer L. (2009). Mediterranean Sea Surface Warming 1985–2006. Clim. Res..

[10] Kuleli T., Bayazit S. (2020). Summer season sea surface temperature changes in the Aegean Sea based on 30 years (1989–2019) of Landsat thermal infrared data. Environ. Monit. Assess..

[11] Pavlidis P., Karakasi M.-V. (2019). Greek land borders and migration fatalities—Humanitarian disaster described from the standpoint of Evros. Forensic Sci. Int..

[12] Garcia Borja A., Abdelazim M. (2021). The Situation of Migrant Deaths in the Mediterranean in 2021.

[13] Helldén D., Andersson C., Nilsson M., Ebi K.L., Friberg P., Alfvén T. (2021). Climate change and child health: A scoping review and an expanded conceptual framework. Lancet Planet. Health.

[14] Ducharme M.B., Lounsbury D.S. (2007). Self-rescue swimming in cold water: The latest advice. Appl. Physiol. Nutr. Metab..

[15] Giesbrecht G.G., Arnett J.L., Vela E., Bristow G.K. (1993). Effect of task complexity on mental performance during immersion hypothermia. Aviat. Space Environ. Med..

[16] Taylor L., Watkins S.L., Marshall H., Dascombe B.J., Foster J. (2016). The Impact of Different Environmental Conditions on Cognitive Function: A Focused Review. Front. Physiol..

[17] Martin K., McLeod E., P’eriard J., Rattray B., Keegan R., Pyne D.B. (2019). The Impact of Environmental Stress on Cognitive Performance: A Systematic Review. Hum. Factors J. Hum. Factors Ergon. Soc..

[18] Muller M.D., Gunstad J., Alosco M.L., Miller L.A., Updegraff J., Spitznagel M.B., Glickman E.L. (2012). Acute cold exposure and cognitive function: Evidence for sustained impairment. Ergonomics.

[19] Thomas J.R., Ahlers S.T., House J.F., Schrot J. (1989). Repeated exposure to moderate cold impairs matching-to-sample performance. Aviat. Space Environ. Med..

[20] Nocentini U., Giordano A., Di Vincenzo S., Panella M., Pasqualetti P. (2006). The Symbol Digit Modalities Test—Oral version: Italian normative data. Funct. Neurol..

[21] Hodgdon J.A., Hesslink R.L., Hackney A.C., Vickers R.R., Hilbert R.P. (1991). Norwegian military field exercises in the arctic: Cognitive and physical performance. Arct. Med. Res..

[22] Pepper R.L., Kennedy R.S., Bittner A.C., Wiker S.F., Harbeson M.M. (1985). Performance Evaluation Tests for Environmental Research (Peter): Code Substitution Test. Percept. Mot. Ski..

[23] Suhr J.A., Stewart J.C., France C.R. (2004). The Relationship between Blood Pressure and Cognitive Performance in the Third National Health and Nutrition Examination Survey (NHANES III). Psychosom. Med..

[24] Wright K.P., Hull J.T., Czeisler C.A. (2002). Relationship between alertness, performance, and body temperature in humans. Am. J. Physiol. Integr. Comp. Physiol..

[25] Du Bois D., Du Bois E.F. (1989). A formula to estimate the approximate surface area if height and weight be known. 1916. Nutrition.

[26] Nieman D.C., LaSasso H., Austin M.D., Pearce S., McInnis T., Unick J. (2007). Validation of Cosmed’s FitMate™ in Measuring Exercise Metabolism. Res. Sports Med..

[27] Tanaka H., Monahan K.D., Seals D.R. (2000). Age-predicted maximal heart rate revisited. J. Am. Coll. Cardiol..

[28] Barwood M.J., Corbett J., Green R., Smith T., Tomlin P., Weir-Blankenstein L., Tipton M.J. (2012). Acute anxiety increases the magnitude of the cold shock response before and after habituation. Eur. J. Appl. Physiol..

[29] Eglin C.M., Tipton M.J. (2004). Repeated cold showers as a method of habituating humans to the initial responses to cold water immersion. Eur. J. Appl. Physiol..

[30] Hayward J.S., Eckerson J.D., Collis M.L. (1975). Effect of behavioral variables on cooling rate of man in cold water. J. Appl. Physiol..

[31] Cheung S.S., Ainslie P.N. (2022). Chapter 6: Cold Water Immersion. Advanced Environmental Exercise Physiology.

[32] Šrámek P., Šimečková M., Janský L., Šavlíková J., Vybíral S. (2000). Human physiological responses to immersion into water of different temperatures. Eur. J. Appl. Physiol..

[33] Weiß M., Hack F., Stehle R., Pollert R., Weicker H. (1988). Effects of Temperature and Water Immersion on Plasma Catecholamines and Circulation. Int. J. Sports Med..

[34] Clark K.L., Noudoost B. (2014). The role of prefrontal catecholamines in attention and working memory. Front. Neural Circuits.

[35] Friend A.T., Balanos G.M., Lucas S.J. (2019). Isolating the independent effects of hypoxia and hyperventilation-induced hypocapnia on cerebral haemodynamics and cognitive function. Exp. Physiol..

[36] Watanabe A., Matsuo K., Kato N., Kato T. (2003). Cerebrovascular Response to Cognitive Tasks and Hyperventilation Measured by Multi-Channel Near-Infrared Spectroscopy. J. Neuropsychiatry Clin. Neurosci..

[37] Tipton M.J., Stubbs D.A., Elliott D.H. (1991). Human initial responses to immersion in cold water at three temperatures and after hyperventilation. J. Appl. Physiol..

[38] Tipton M.J., Golden F.S.C., Higenbottam C., Mekjavic I.B., Eglin C.M. (1998). Temperature dependence of habituation of the initial responses to cold-water immersion. Eur. J. Appl. Physiol. Occup. Physiol..

[39] Tipton M.J., Mekjavic I.B., Eglin C.M. (2000). Permanence of the habituation of the initial responses to cold-water immersion in humans. Eur. J. Appl. Physiol..

[40] Petrass L.A., Blitvich J.D. (2014). Preventing adolescent drowning: Understanding water safety knowledge, attitudes and swimming ability. The effect of a short water safety intervention. Accid. Anal. Prev..

[41] Willcox-Pidgeon S.M., Franklin R.C., Leggat P.A., Devine S. (2020). Identifying a gap in drowning prevention: High-risk populations. Inj. Prev..

